# Identifying the Impacts, Obstacles and Information Barriers for Parents of Children Living With Genetic Neurodevelopmental Disorders: A Qualitative Study

**DOI:** 10.1111/hex.70340

**Published:** 2025-07-03

**Authors:** Karen J. Low, GenROC Consortium, Suzanne Alsters, Ruth Armstrong, Tazeen Ashraf, Queenstone Baker, Meena Balasubramanian, Diana Baralle, Jonathen Berg, Ishita Bhatnagar, Marta Bertoli, Thomas Boddington, Moira Blyth, Catherine Breen, Helen Brittain, Lisa Bryson, Jenny Carmichael, Emma Clement, Tessa Coupar, Anna de Burca, Cristina Dias, Fleur S. van Dijk, Abhijit Dixit, Alan Donaldson, Andrew Douglas, Jacqueline Eason, Nour Elkhateeb, Sahar Elkady, Fayadh Fauzi, Elaine Fletcher, Helen V. Firth, Nicola Foulds, Caroline Furnell, Andrew E. Fry, Laura Furness, Jennifer Gardner, Gabriella Gazdagh, Merrie Gowie, Abigail Green, Asma Hamad, Rachel Harrison, Verity Hartill, Lizzie Harris, Eleanor Hay, Jenny Higgs, Jonathon Hoffman, Simon Holden, Daniela Iancu, Rachel Irving, Vani Jain, Rosalyn Jewell, Diana Johnson, Gabriela Jones, Beckie Kaemba, Arveen Kamath, Ayse Nur Kavasoglu, Tabassum Khan, Mira Kharbanda, Sophie King, Usha Kini, Alison Kraus, Ajith Kumar, Katherine Lachlan, Neeta Lakhani, Wayne Lam, Anne Lampe, Abigail Lazenbury, Harry Leitch, Helen Leveret, Samuel Liebert, Jessica Maiden, Anirban Majumdar, Alison Male, Alisdair McNeil, Ruth McGowan, Holly McHale, Catherine McWilliam, Jonathan Memish, Lara Menzies, Radwa Mohamed, Tara Montgomery, Oliver Murch, Fiona Osborne, Michael Parker, Caroline Pottinger, Vijayalakshmi Ramakumaran, Ruth Richardson, Lisa Robertson, Alison Ross, Claire Searle, Wofah Selah, Resifina Seyara, Charles Shaw‐Smith, Suresh Somarathi, Charlotte Stanley, Edward Steel, A Stewart, Helen Stewart, Kerra Templeton, Riya Tharakan, Madeline Tooley, Mohamed Wafik, Emma Wakeling, Elizabeth Wall, Amy Watford, Patricia Wells, Emily Woods, Louise Wilson, Georgia Treneman‐Evans, Sarah L. Wynn, Jenny Ingram

**Affiliations:** ^1^ Centre for Academic Child Health Bristol Medical School University of Bristol Bristol UK; ^2^ Department of Clinical Genetics UHBW NHS trust Bristol UK; ^3^ Unique Rare Chromosome Disorder Support Group Oxted UK

**Keywords:** distress, genomics, parent, rare

## Abstract

**Background:**

A genetic neurodevelopmental disorder (GND) impacts all aspects of a child's and family's life. GNDs are rare; most have limited natural history data. We aimed to understand the impacts, obstacles, information barriers and coping strategies developed through parents' experience of receiving and living with a child's diagnosis.

**Design and Participants:**

This analysis is part of the UK multicentre observational study of children with rare GNDs (GenROC). We conducted 17 semi‐structured online interviews with parents of children with GNDs (aged 0–15 years) from November 2023 to March 2024. Data were analysed following the principles of thematic analysis.

**Results:**

We identified five themes. (1) *Impact on the family around a genetic diagnosis*: Distress begins well before a diagnosis is received; there is an impact upon the receipt itself and the ongoing impact on the family thereafter. (2) *Impact of uncertainty, lack of data and ‘rareness’*. The experience of parenting when so little is known about your child's condition. (3) *Relationships with health professionals*. Positive where parents are empowered and feel part of the team; negative where parents feel not heard/believed due to a professional lack of expertise/understanding. (4) *Parent mental health*: GNDs can be a significant burden to family life. The need to advocate for services has a negative impact. Feelings of isolation through rareness. (5) *Coping strategies and factors that help*: Support/Facebook groups are considered highly beneficial. Parents develop new positive identities, including that of advocate, professional and educator.

**Conclusions:**

GNDs represent a major challenge for families, clinicians and service providers. Distressed parents are struggling to cope with challenges and suffer from poor mental health. Psychosocial support, better signposting and health professional education may help.

**Patient Contribution:**

Patient Participant Involvement group (comprising five mothers and one father of children with varying GNDs, one young person with a GND, and one genetics family charity representative) contributed to topic guide development and methodology and provided feedback on results.

## 1 Background

The reducing cost of Whole‐Genome Sequencing (WGS) has resulted in more children being diagnosed with rare genetic neurodevelopmental disorders (GNDs) than ever before in the United Kingdom, often within non‐specialist settings, and sometimes with the diagnosis being received at a very early age [1, 2]. Clinical decision‐making regarding whether a child (living in England) can or should be tested depends on the child meeting specified testing criteria as determined by a nationally agreed document, ‘the National Genomic Test Directory’ [3]. For example, to qualify for ‘paediatric disorders’ testing, a child would need to have ‘Congenital malformations and/or dysmorphism suggestive of an underlying monogenic disorder’. For children in the United Kingdom, this testing is provided as part of NHS care (parents do not need to pay); however, testing can only be requested by a clinical geneticist or a specialist paediatrician.

When assessing the impact of a genetic diagnosis, previous studies have focussed on decisional regret in those groups [4, 5] who received a genetic diagnosis as compared to those whose did not. Some of the studies have focussed on the impact on parents whose children have genetic testing whilst critically unwell, typically in neonatal intensive care [5, 6]. However, there are limited data documenting parents' and carers' (from here on collectively referred to as parents) views on their experience and levels of support as they then go on to navigate the landscape of having a child with a genetic condition. There are a small number of studies that report the lived experience with a very specific genetic disorder and age group [7, 8]. Insight into parent's experiences of receiving genomic diagnosis alongside their lived experience as a consequence of said diagnosis is needed to inform policy and clinical practice as WGS continues to be implemented widely.

We aimed to understand the lived experience of parents of children with GNDs to identify the needs, obstacles and barriers for families relating to their child's genetic diagnosis. Our primary research question was to understand the parent's experience of receiving the diagnosis, the impact of the diagnosis on the parents and family and how they navigate their child's care as a consequence. These results may inform practice and underpin future recommendations for improving family support and clinical practice.

## 2 Methods

### 2.1 Study Design

This qualitative study is part of the national multicentre study ‘Improving the clinical care for Genetic Rare disease: Observational Cohort Study’ (GenROC: REC22/EM/0274) [5]. We aimed to understand the lived experience of parents of children with GNDs to identify the needs, obstacles and barriers for families relating to their child's genetic diagnosis. To understand their experience, the first author undertook semi‐structured interviews with parents of children with known GNDs aged 0–15 years. Interviews were based on a semi‐structured topic guide that had undergone ethical approval as part of the wider approval process for the GenROC study [9]. The topic guide was informed by the relevant literature [4, 5, 6, 7, 8, 10, 11, 12, 13, 14, 15, 16] and developed with professionals and stakeholders in the area of rare disease, as well as the study's Patient Participant Involvement (PPI) group. The topic guide was presented to the PPI group (comprising five mothers and one father of children with GNDs, one young person with a GND, and one genetics family charity representative), who provided comments on the structure and content as well as the planned interviews. Particular feedback included that the guide needed to allow for time for parents to revisit their diagnostic journey and also that parents should be allowed to be interviewed together if they wished to do so. PPI strongly recommended online interviews for practical acceptability.

The interviewer was a senior clinical geneticist who has had training in qualitative research methods and no prior contact with the participants. All interviews took place online with participants in their homes using the video conferencing platform, Zoom. A child was present in one interview.

The topic guide follows a predefined structure but allows additional questions and emerging issues in the context of GNDs. This guide includes (1) an introduction of the study and interviewees, then focussed on (2) the families' pathway leading up to and during the diagnosis, (3) daily living with a GND and its impact on navigating healthcare, (4) what information families have been missing and how they have looked for this information and accessed it including areas of support and (5) if and how they utilise digital health and social media in this respect and (6) a chance for parents to discuss anything else that they felt was important (see Supporting File 1).

### 2.2 Participants and Sampling

Inclusion and exclusion criteria for participation in the interview were as for enrolment into GenROC [6], which were children aged from 6 months to 16 years with a confirmed diagnosis in a set list of GNDs living in the United Kingdom.

Parents completed consent to participate in the GenROC study, and of these, 91% agreed to being contacted about an interview.

**Figure 1:**
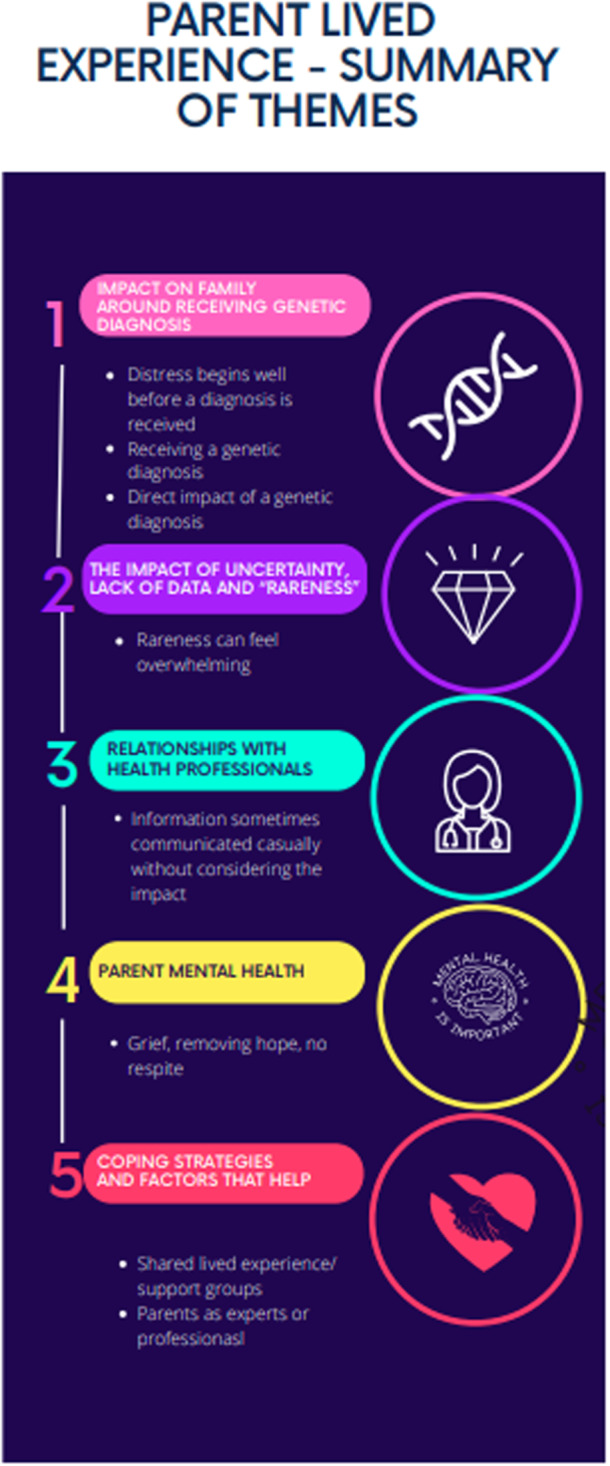
Summary of themes.

We estimated that we would aim to interview 10–20 parents and purposively sampled participants from families recruited to GenROC to ensure maximum variation across the following categories: (1) postcode Indices of Multiple Deprivation (IMD) centile; (2) type of genetic disorders (Figure 1); (3) UK geography; (4) ethnicity; (5) children of differing ages; (6) foster and adoptive parents; (7) parents advocates, leads of support groups or charities; (8) fathers were actively recruited through the invite email which specifically highlighted that we were keen to hear from fathers if possible (as well as mothers), which increased our number of fathers included by four. We agreed that parents could request to be interviewed together as a couple, which enabled some of the fathers to take part and empowered them to be part of the discussion. The sampling factors were intentionally chosen to allow for demographic and diagnostic variation in the sample. We also used our clinical insight and expertise to select participants with specific characteristics, such as parents who are advocates/charity leads and foster carers, as we anticipated that they would provide strong dialogue to satisfy our study aims.

Parents were invited to take part by email. They were informed about the study using a Parent Information Leaflet, which had undergone ethical approval as part of the wider GenROC study [6]. Parents were required to complete an interview‐specific consent form before the interview. Appointments dates and times for the interview were agreed upon via email. No participants refused to participate, nor did any drop out.

### 2.3 Data Collection

The interviews were conducted between November 2023 and March 2024. Interviews were conducted by the lead author, who is the chief investigator of the GenROC study. All interviews were recorded, transcribed verbatim and anonymised and combined with the interviewer's field notes for analysis. Transcriptions were imported into NVivo qualitative data management software (Release 1.71) for coding and data management. Using the concept of information power to inform the sample sufficiency, we expected that using a case analysis strategy with specific participants who would provide strong dialogue would satisfy our relatively narrow study aims [17]. After eight parents were interviewed, the process was paused to review the data and to develop and refine the coding matrix. We determined that we would continue with interviews until no new code categories were being generated and no new themes were created [18, 19]. No repeat interviews were undertaken.

### 2.4 Reflexivity Statement

K.J.L. (interviewer) is a senior clinical geneticist with particular expertise in caring for children with GNDs and with a background in paediatrics. She is also a mother of two neurodiverse children and has a rare disease herself. G.T.E. (second coder) is a non‐clinical, experienced qualitative researcher with no genetics expertise. Both live in Bristol, the United Kingdom. G.T.E. is an experienced psychological and behavioural researcher and has conducted many qualitative studies and provided guidance for K.J.L., as this was her first qualitative study. K.J.L. has conducted multiple clinical studies into GNDs.

### 2.5 Data Analysis

Reporting of data has been performed according to COREQ guidelines [20]. Data were analysed following the principles of inductive thematic analysis based on an interpretive/constructive epistemological perspective to explore the lived experience of parents [18, 19, 21]. An iterative approach was used to develop an initial codebook informed by the aims of the study (deductive component). The first eight transcripts were coded by the first author, and additional codes that were not covered by the initial codebook were then added (inductive component). 20% of the transcripts were double‐coded by the second author. All discrepancies were reconciled through consensus. The first author discussed her impressions and assumptions with the second coder reflexively. NVivo software was used to group codes, and these were refined following discussions between the authors. Methodological oversight was provided by the final author, who was also a non‐clinical qualitative researcher.

## 3 Results

All sampled participants accepted the invitation, and 13 interviews were conducted with a total of 17 parents whose characteristics are summarised in Table 1. Interviews lasted an average of 46 min with the shortest being 27 min (single parent interview) and the longest being 58 min (two‐parent interview).

**Table 1:** Table of sample characteristics.

Sample characteristics	Parents (*N* = 17)	Percentage of sample
	*N*	%
Gender of parent:		
Female	12	70.5
Male	5	29.5
Family structure:		
Cohabiting/Married	14	82.4
Single parent	3	17.6
Age of parent:		
20–29	2	11.8
30–39	8	47.0
40–49	6	35.3
50–59	1	5.9
Ethnicity of parent:		
White	13	69.9
Asian	3	17.6
Black, Caribbean or African	1	12.5
Employment of parent:		
Professional	5	29.4
Manager	2	11.8
Service	3	17.6
Technical	1	5.9
Full‐time carer	6 of these 2 were foster carers	35.3
IMD centile:		
1–2	3	17.6
3–4	4	23.5
6–8	6	35.3
9–10	3	17.6
Not available	1	
Age of child	(*N* = 13)	
0–2	2	15.4
3–4	5	38.5
5–6	2	15.4
7–9	4	30.7
Genetic condition/Gene**	(*N* = 13)	
CHD2	1	7.6
DYNC1H1	1	7.6
SCN1A	1	7.6
SOX5	1	7.6
OPHN1	1	7.6
HUWE1	1	7.6
DYRK1A	1	7.6
GRIN2B	1	7.6
CASK	1	7.6
EBF3	1	7.6
SLC6A1	1	7.6
KCNQ2	1	7.6
CACNA1	1	7.6

** Genetic condition/gene refers to the gene in which there is an abnormality that has caused the child to have a neurodevelopmental disorder. These conditions include a variable spectrum of developmental delay and intellectual disability; growth and feeding abnormalities; seizures; autistic spectrum disorder and variable other physical features—the particular set of features will vary depending on the gene.

The five themes identified are shown in Figure 1. Themes are presented with illustrative quotes in the text, and further quotes are marked in brackets such as (Q1) in Table 2 and identified by participant number and indicating whether ‘mother or father’.

**Table 2:** Further quotes from participants organised by themes.

Theme	Quote number	Quote
Impact on the family around receiving a genetic diagnosis		
Distress	1	‘Then I went to the doctor with him, the paediatrician, and the first paediatrician was actually quite awful, don't think it was his fault, he was clearly having a bad day, and he was a locum,' and he said, ‘I've never heard a mother complain to me that the baby doesn't cry before.’ #12, mother
	2	I said, ‘I'm a bit worried he's having seizures.’ ‘No, no, babies do that it's fine, don't worry,’ and I am a paediatric nurse, I worked in a tertiary centre in a ward with lots of children with epilepsy, so it was like, 'am I seeing what I think I'm seeing which are really subtle seizures, or am I being super paranoid because that's my work environment?’ #11, mother
Receiving diagnosis	3	‘Well we don't need that operation, but it's because her brain's not growing.’ 'As a parent getting the news that your child's brain isn't growing on the telephone from a junior doctor that was pretty devastating.’ #10, mother
Impact of diagnosis	4	‘But now we can go in and say, “He's got this,” and all encompassing, rather than us having to sit down, and we do still go through the list of make sure they're clear on what it is, but before they were just … yeah it wasn't … it's quite a long conversation if you go in a room and spend … we would spend 10 or 15 min of a GP appointment… paediatrician appointment just stating the complications, and then we would move on to the actual issue we've got. Whereas now we can say this and then focus on the key bits.’ #7, father
	5	‘I think having the diagnosis then we could find our group, and even in our group there's differences. But now we can … we've got families on Instagram and Facebook, and we can see what a day in the life for their family, … going back to the progression and what the future could possibly hold for [], you get that satisfaction, and you get the camaraderie of being part of a group that can work together.’ #6, mother
Impact of uncertainty, lack of data and rareness		
Uncertainty	6	‘At the time I think the consultant didn't seem to know any other families in the UK … and it's no disrespect to anyone but it was like this is it, this is the diagnosis, and with the best will in the world there are so many of these rare conditions that the parents end up becoming more expert in it than the [doctor] … she was just genuinely like, “There's so many of these things.”' #12, mother
	7	‘and then it's like because he had a diagnosis, all these tests started stopping, it was like just part of his syndrome, and we were like but what if it's not? And it just got more frustrating, even though we have the answers to what was wrong with him, it got more frustrating then because it were just like oh well he's got a diagnosis now … they just blame it on his diagnosis all the time.’ #1, mother
	8	‘just going down a tunnel of I want to read up everything about this now, and just being up at early hours just reading through all these articles, and you should be sleeping, but you just want to do everything you can to help really.… It's difficult finding factual information that were credible sources.’ #6, mother
Relationship with health professionals		
	9	‘Yeah, and then they look at you and then sometimes shut you down and tell you, you don't know what you are talking about. The gene doesn't do that kind of thing and you're having to fight.’ #4, mother
	10	‘They don't know how to live with a child with these conditions, and they don't know, they can't understand … but you and your wife haven't slept for two weeks … but for them they'll just skip over a fact because they want to move onto the next thing.’ #7, father
	11	‘Because when we saw a better paediatrician, luckily, who did spend an hour and a half with us the first appointment which now that I go see him fairly regularly I know that was a big chunk of his morning clinic that day, and he literally just let me talk, and he said, “is there anything else? Is there anything else you are worried about?”' #12, mother
Parent mental health		
	12	‘it's almost like they assume that you're being an over‐anxious parent first, and then you have to prove them differently, it's not the other way round … and that can actually make you mad, it actually can. I know a lot of parents and carers that really have had very bad mental health from it.’ #5, mother
	13	‘Sometimes the behaviour can be so bad that it really is the biggest burden on family life…. It means that we can't go out alone on the weekends with both of them, so you have to always have two adults. They're getting older, they're both mobile, they both are fully unaware of danger, they don't listen, they don't stop, they don't look back, they don't care if they've left you or not left you. They will run out in the road, they will walk off with strangers. They are not in tune with any of that, and if you've got one having a full on crazy meltdown that might last up to 40 min, and you've still got another one that you're trying to hold onto as well, you just can't do that on your own.’ #11, mother
Coping strategies and factors that help		
Shared lived experience and support groups	14	‘They're staring at you because it's a window into their future, and because this is … people hate the unknown. So it's almost like if they can see that you're 16 and that you're at school, and that you've passed your IT exams, that gives parents of younger kids hope.’ #12, mother
	15	‘so we went on to have another child, and it was a really big decision, and I don't think we would have had the guts to do that if we hadn't seen these other families on Facebook, and it's like that showed us that it was possible … just seeing that there was all these other families on there where they'd gone on to have other children, and I think that influenced us quite a lot in terms of possibilities.’ #15, father
Parents as experts or professionals	16	‘But he wanted to know the co‐ordinates so he could then go and research and … because he found it really useful to … was helpful for him so that he could then go and learn all that, and learn where the terminus was in the gene that meant that she didn't make the protein, which meant that she then has these complications.’ #16, mother

### 3.1 Impact on the Family Around Receiving a Genetic Diagnosis

#### 3.1.1 Distress Begins Well Before a Diagnosis Is Received

Most parents described worrying about their child for some time before receiving a diagnosis due to antenatal abnormalities, medical concerns or unusual features or behaviours in the child, or a failure to meet expected milestones (Q1).

Nonetheless, it often takes multiple visits and involvement of many healthcare professionals before a child is recognised as possibly having a genetic diagnosis. Parents feel unheard, anxious and alone in their concerns, but their gut feeling that something is wrong leads them to persist for answers and push for genetic testing despite being told their child is healthy, often by more than one professional. Because they are certain that there is something wrong, they start researching possible genetic conditions that may fit with their child's physical features and medical conditions (Q2).

‘Please just tell me what's wrong with my baby? And they were like we don't know, we need these results back.’

#1, Mother

Once genetic testing is activated, parents universally describe a difficult period of waiting for the results. One mother described waiting in an outpatient's waiting area for 4 h in the hope she would be seen early because she was so desperate to hear the result.

#### 3.1.2 Receiving a Genetic Diagnosis

Parents describe receiving devastating information delivered in a casual or informal manner (Q3). Professionals deliver diagnoses in differing settings and formats. Sometimes this was only over the phone or video call (some received diagnoses during Covid). Telephone conversations do not always allow parents sufficient time to digest the information. Complicated concepts such as ‘misfiring potassium channels’ may be difficult to understand, which can be exacerbated over the phone. This can impact parents' own ability to research the condition later on.

Some parents were given the diagnosis initially by a paediatrician but then told they needed to be referred to a geneticist for further discussion. This additional wait heightens distress to parents who reported that they would have found it easier without a time lapse.

Parents also describe the difficulty in receiving a diagnosis and complex information during an appointment in which they are also expected to manage their child's needs.

‘Because in an appointment with [child], child is 100 miles an hour, and it feels like when I come out of appointment it feels like I've run a marathon. Because I'm trying to talk to the doctor and received information whilst stopping her cracking her head open.’

#17, mother

#### 3.1.3 Direct Impact of the Genetic Diagnosis

Parents all report that the genetic diagnosis had been helpful in removing their guilt that they may be somehow responsible for their child's challenges. It enables them to apply for support for their child, including benefits such as Disability Living Allowance or additional educational support in the form of an Educational Health Care Plan (EHCP). It can guide clinicians regarding tailored treatment and monitoring plans for their child (Q4). Families can access condition‐specific support groups and can get some idea of what to expect for their child in the longer term. Parents report that the diagnosis provides an all‐encompassing label that validates their requests with professionals, thereby reducing their need to fight for help. They are grateful that it might prepare their child better should they go on to have a child of their own (Q5).

### 3.2 The Impact of Uncertainty, Lack of Data and ‘Rareness’

Rareness can feel overwhelming, especially when the number of children affected worldwide is very small.

‘Sometimes I sit and think maybe I wish he did just have Down's syndrome, and then everyone knows it [rather] than having X [and] no one knowing it’.

#1, mother

Professionals who are giving the diagnosis often know very little about the condition and simply direct parents to Facebook support groups. Professionals blame the large number of rare conditions for their lack of expertise (Q6).

Some parents are anxious that having a genetic diagnosis can result in a child's challenges being incorrectly attributed to that condition and not being correctly investigated (Q7).

Parents, therefore, need to pursue information that adds to their mental load. Some parents reported frustration around the lack of detail provided to them at diagnosis with particular respect to their child's specific genetic variant, which then hampers their self‐directed research efforts (Q8).

Detailed natural history data for GNDs are often limited, with the pace of new data acquisition slow. This can lead to parental frustration and worry when their child develops a new problem and limits parents' ability to plan and manage expectations for their family and the future. Recurrence risks quoted to parents were unclear or differed between health professionals and the support groups/research. A parent described the devastation of having a second affected child following genetic counselling of a low recurrence risk. The general population has a limited understanding of GNDs. This places an additional burden of explanation on parents to extended family, friends and wider community. Some describe the frustration of people trying to suggest they have lived experience when they do not.

### 3.3 Relationship With Health Professionals

Professionals can communicate devastating information casually without seeming to consider the impact on a parent.

‘Anyway, he did phone, and I was all by myself, and he was very jubilant in that he'd been right. So, it was a, “Yes I was right, I'm amazing, she had X,” which obviously if Google, X only has a very short life expectancy.’

#10, mother

Parents are required to advocate for their child endlessly and feel continuously doubted by professionals which can be extremely hard, tiring and upsetting. Foster carers can face a higher threshold of belief by professionals who question how ‘well’ they know the child.

Parent‐professional hierarchies and professional egos result in difficult interactions when parents present new data or question the clinician's opinion. Parents perceive that some professionals (particularly hospital consultants) don't like to be told what to do by a parent (Q9).

Parents may hear about possible treatments or therapies that children with the same GND are on, and this may be through their own research or through social media groups and connecting with other families. This may suggest to them their child should be receiving a treatment or therapy which they have not been able to access. Service pressures result in parents having to be more demanding than they would wish to be which is exhausting, and busy clinical environments can lead to a perceived lack of empathy (Q10).

Having a good relationship with the child's key paediatrician was reported as being an important support. Parents highly prized an egalitarian relationship with a doctor in which they were empowered to bring new information and questions and were listened to without judgement with time and space to talk (Q11).

Parents reported that their own education and work background sometimes impact positively or negatively on health professional attitudes towards them with examples being a biology teacher (who felt she was treated more as an equal) and a shop worker (who felt talked down to), respectively.

Parents would like to have a named clinical expert for their child's genetic condition who can coordinate care nationally; however, this is very variable as some conditions do not have one and for many the ‘expert’ is in another country. Parents may travel to attend the international family conference at considerable personal expense which would not be possible for many families and can be impossible with complex childcare needs and a lack of a wider family network to help. However, those who have accessed an ‘expert’ reported that it reduced their anxiety and resulted in them feeling better informed and able to support their child.

### 3.4 Parent Mental Health

Parents describe the burden of living with GND as all encompassing, and they experience a period of grief around the diagnosis. Some described the unfairness or ‘bad luck’ of having a genetic condition. Many parents spoke about the finality of a genetic diagnosis and how ‘it is for life’ and can remove the hope of a child getting better or being ‘normal’.

‘Then like a period of mourning really. You have to let go of the future you thought was going to happen, and you adapt … you're a parent to a severely child with a disability, and it touches every area of everything you do’.

#15, father

Many parents described that having a child with GND along with lack of support has resulted in a significant deterioration in their mental health. This is exacerbated by a constant need to fight with professionals to access care and support for their child; in one case, this battle led to a social services referral. Professionals sometimes assume parents are overanxious which can cause an additional impact (Q12).

Managing children's challenging or unsafe behaviour is particularly difficult for families. Parents need to be constantly vigilant and cannot get respite, placing a huge demand on them daily (Q13).

#### 3.4.1 Coping Strategies and Factors That Help

##### 3.4.1.1 Shared Lived Experience and Support Groups

Shared lived experience with other families is the most highly reported coping strategy. Parents use the group as a sounding board regarding new concerns and use the responses to determine a course of action.

Groups allow parents to assess for themselves where their child fits in the spectrum of the condition and what the future may hold in a way that isn't possible using ‘dry’ scientific literature. Lack of natural history data and variability of phenotype expression lead parents to worry about what the future holds for their child. They look to support groups for examples of older individuals. Finding examples of individuals who have achieved certain milestones in education or independence provides parents with hope (Q14).

‘Because they're having the exact same experience and getting to understand beyond what the papers say about the spectrum of the disease, and what people go through, and what their children go through, and how they present across the lifespan. There's several adults in the group, so it gives us a little bit of a glimpse what the future might look like.’

#11, mother

Parents consult the groups to get practical information about toys, types of mobility support aids and days out, among other things. They develop ‘good friends’ in the groups and report sending and receiving birthday cards/presents and gifts when their child is admitted to the hospital.

Whilst most of the groups interacted predominantly online, one UK group organised an annual family weekend at a holiday park, part funded by fundraising grants. A parent reported how beneficial these weekends were in terms of providing a support network, including for other siblings. The experience of seeing other families who had gone on to have another (unaffected) child influenced their subsequent reproductive decisions (Q15).

##### 3.4.1.2 Parents as Experts or Professionals

Parents consistently want and need to become experts in their child's rare condition to allow them to access appropriate health, education and support. As a consequence, some parents, particularly those who have a scientific background or interest, enjoy the process of researching the genomic science of their child's condition. For some, particularly fathers, this allows them to feel that they are actively doing something to help in the context of a situation in which they frequently feel helpless (Q16).

A smaller number of parents evolved to become peer supporters and provide informal advice to less well‐informed parents or parents of newly diagnosed children. This role as ‘advisor’ provides purpose and self‐esteem as well as the positive benefits of being able to help other families. Some parents in our study have set up foundations and charities, which opened up a whole new career for them.

‘But I was floating around aimlessly for years, I didn't know what to do because I had to give up my job, because I was a teacher, and then I was just caring for [daughter], and I'm not really very good at that kind of aspect. I would never have been a carer, it's not something I would ever have chosen to do, so I wasn't getting fulfilment out of just staying at home and caring for her…. So I set up the website actually before I set up the charity.’

#10, mother

Foster parents are professionals who choose to look after children with challenging, complex needs and describe themselves as ‘project managers’. They describe great fulfilment from their role, both personally and professionally. Other parents describe an evolution from parent as expert to advocate and professional. Some have set up condition‐specific websites, foundations and charitable organisations, dedicating considerable time and effort to this, often on a voluntary basis. They describe linking in with professional groups, attending conferences and making presentations in multiple settings, enabling them to continue to develop personally and professionally, and giving them fulfilment in a role other than parent with considerable positive impact.

Parents view the opportunity to take part in genetic research studies as a positive way to improve their chance of getting more data on their child's condition as well as valuing the possibility of helping other families in the future.

## 4 Discussion

This study highlights the significant challenges faced by parents of children with GNDs and their families. Parents experience considerable distress throughout all stages of their child's care pathway and diagnostic journey, including accessing services and interacting with professionals. This distress can result in a deterioration in parents' mental health. Optimising care to reduce distress factors could improve parents' capacity to cope, which could potentially have a positive impact on mental health.

### 4.1 Parent's Experience of Receiving the Diagnosis and the Impact of the Diagnosis on the Parents and Family

The diagnostic wait is difficult for families [10]. Access to genomic testing has improved in the last decade, but many parents still describe a period of time in which they knew something was not right with their child, but were dismissed by healthcare professionals, or they could not access the appropriate service. Even in cases where a diagnosis was made at a young age, parents report the lag time leading up to that diagnosis as being distressing. Frustration and distress associated with the diagnostic odyssey have also been reported in parents interviewed as part of an undiagnosed diseases programme [11]. Nonetheless, there is clear evidence in the literature and from our findings that parents consider a diagnosis to be enormously beneficial as it gives a reason ‘why’ and opens doors to services and support [4, 5, 10, 11, 12, 14, 22, 23]. Distress appears to be unavoidably related to the period of time when parents do not have an explanation or answers for their child's symptoms [4, 11]. It may be that some of this distress is not avoidable, as much of it relates to having a child who has problems, often unexpectedly. However, attempts to reduce waiting should be prioritised at all stages.

**Figure 2:**
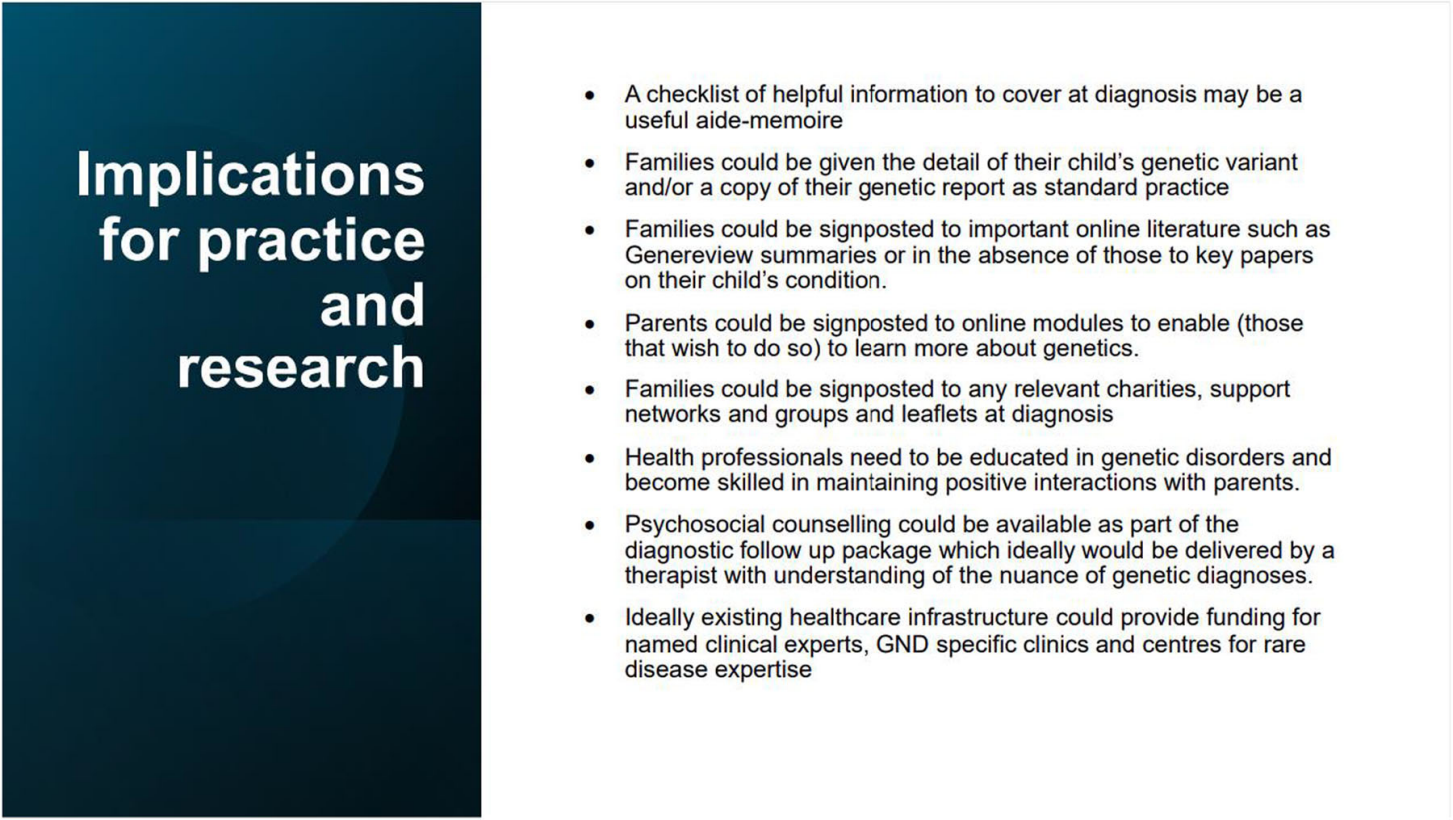
Implications for practice.

The lack of information in GNDs leads to distress and worry [5, 10], and this was apparent in the current findings. Most of the time, it is not possible to predict accurately where their child would fall in the long term compared to others, raising anxiety and making it difficult to plan. Raising the profile of adults with these GNDs and allowing them to become mentors and advocates (as has been done for another rare disorder) [7] may be a mutually beneficial strategy going forward as more children who benefited from exome sequencing diagnoses grow up (Figure 2).

Parents may feel alone despite receiving a diagnosis [4, 5, 12, 14]. Shared lived experience and peer support are beneficial for many parents [12]. This allows them to ask questions and find out information from other parents, including finding out about resources available for their children [8, 12, 24]. This was echoed by some of the parents in our study who reported that peer support groups can give you useful insight into options they had been unaware of [25]. Parents of GNDs can become socially isolated due to lack of understanding from society, complexity of their child's needs [12, 13, 26, 27] due to worry and shame related to their child's behaviour and people not being able to understand that their child had a complex underlying diagnosis [28]. A virtual peer support group circumvents these barriers and reduces social isolation [25, 29, 30]. The diagnosis discussion should include information about support groups and ideally signpost families to an appropriate group so that they are aware of these from an early stage (Figure 2).

A genetic diagnosis is not the same as any other paediatric diagnosis [8, 10, 14, 23]. A mixed‐methods study of the lived experience of parents of children with CDKL5 deficiency disorder cited grief and an associated grieving journey in the context of receiving the diagnosis as a nearly universal theme amongst parents [8]. Our findings highlight that the ‘genetic‐ness’ of the diagnosis had very specific impacts on parents. For instance, parents felt that the genetic diagnosis means that the condition can't be reversed, and it's not going to go away, and it removed the hope of it getting better. These findings were echoed in the Rare Minds 2024 survey [31]. They found that 41% and 85% of respondents felt having or caring for someone with a rare condition had negatively impacted their family relationships and their mental health or emotional well‐being, respectively (Figure 2).

Our findings indicate that for some families and conditions, a genetic diagnosis removes hope [10]. It is important that professionals recognise the way this differentiates this type of diagnosis from others. Psychotherapeutic counselling should be funded specifically for families with genetic disorders, but currently is not available for most and is very rarely offered [31]. 96% of Rare Minds' respondents felt that it was important that mental health professionals had an understanding of rare conditions and how they can impact mental health. This agrees with our findings and we concur with the Rare Minds recommendation [31] that anyone impacted by a rare condition should receive psychologically informed ‘rare aware’ care (Figure 2).

A genetic diagnosis also has an impact on the wider family. The impacts for siblings and family life when having a child with any sort of complex medical condition or neuro‐disability are well documented and include the stressors of managing emergencies, coordinating care, and advocating for your child and fighting the system [8, 10, 15, 16, 27, 28, 32]. Lack of time, socio‐legal difficulties and organisational problems are significant barriers [13, 16, 33]. All parents in our study described some dissatisfaction (of varying proportions) with the healthcare system and professionals. They describe an endless battle to be believed and heard, to access the appropriate care, and to access specialist services. This impacts mental health and reduces their capacity to cope with additional challenges, as well as negatively impacting patient–professional relationships. A centrally coordinated pathway for GNDs with centres of excellence may reduce this burden for families and ultimately improve clinical outcomes (Figure 2).

### 4.2 How Parents Navigate Their Child's Care as a Consequence of the Genetic Diagnosis

Parents develop considerable expertise in managing their child's condition, and this can be both a burden and a source of self‐fulfilment at the same time [8, 13, 16]. Our study shows that parents feel they become experts out of necessity due to the overwhelming need to know more and to try and understand what the future holds. However, some parents take a more active role in becoming experts. For some fathers in our study, becoming an expert allowed them to feel that they were ‘doing something’ active about their situation, which allowed them to cope better with the challenges. Parents should also be signposted to further options for education, explaining basic genetics to minimise their frustration in understanding the information (Figure 2).

It is important for professionals to provide parents with key information about the genetic diagnosis, including the variant details. Peer mentoring may provide a support mechanism in this regard, particularly for parents who wish to become more active as professional advocates [7]. Parents desire a national named clinical expert for their child's rare condition and consider this a priority. Implementation is challenging on a practical level given the number of different rare conditions, funding mechanisms and clinical setups. This point should be considered as a medium‐term strategic priority for rare disease (Figure 2).

### 4.3 PPI Reflections

Our PPI group reported that our findings resonated very strongly with their lived experience. They recognised that professionals are doing their best in often difficult circumstances, but hoped these findings would drive improved practice. A member reflected on the significant burden of being an expert in their child's condition and the fight to be heard.

### 4.4 Strengths and Limitations

Despite our best efforts, the majority of participants were females. A strength of the study is that by agreeing that couples could be interviewed together, we increased the number of fathers who were interviewed. We also noted that whilst some fathers were initially reluctant to speak, and even initially remained off camera in one case, listening to the interview encouraged them to get involved and speak up. However, we acknowledge a potential limitation that interviewing parental dyads may, in some cases, cause one or the other to have a more dominant voice. We intentionally sampled for a wide spread of factors to seek out certain perspectives (e.g., parent advocate and foster carer) and to ensure good sample diversity. Despite this, most, but not all, were white British (reflective of the wider cohort demographic spread), so not all of our findings can necessarily be generalised to all groups. Our cohort only includes children with GNDs. This means that our findings may not be generalisable to all rare diseases. Due to the nature of the study, some of our findings are likely to be relevant to wider patient groups such as those with chronic disease, other forms of disability and neurodiversity. The main interviewer is a senior clinical geneticist, who may represent an insider standpoint in these results. Reflective discussions with the second coder took place to recognise this, and we note that most parents felt empowered to discuss negative experiences with healthcare professionals, suggesting that her professional standing was not a barrier to expressing their views.

## 5 Conclusions and Future Directions

This study provides further insight into parents' experience of receiving a genetic diagnosis, the impact of the diagnosis and how they subsequently navigate their child's care. Whilst genomic testing and diagnoses are now more readily accessible and occurring at a younger age, parents are universally distressed and suffer poor mental health. Parents bravely find coping mechanisms such as advocacy and Facebook groups. Based on our findings, we have suggested practical implications for practice, which include important information and signposting to cover during the diagnosis giving consultation, and an ideal rare disease service provision, which would include nuanced psychosocial counselling.

## Author Contributions

**Karen J. Low:** conceptualisation, investigation, funding acquisition, writing – original draft, methodology, visualisation, writing – review and editing, formal analysis, project administration. **Georgia Treneman‐Evans:** investigation, writing – review and editing, methodology, formal analysis. **Sarah L. Wynn:** writing – review and editing. **Jenny Ingram:** investigation, methodology, writing – review and editing, supervision.

## Disclosure

The views expressed are those of the author(s) and not necessarily those of the NIHR or the Department of Health and Social Care.

## Ethics Statement

The GenROC study received Research Ethics Committee (REC) approval on 15 December 2022 and Health Research Authority approval on 9 February 2023.

## Consent

All participants provided informed consent for the interview as per the GenROC study protocol.

## Conflicts of Interest

The authors declare no conflicts of interest.

## Supporting information

Supporting file 1.

## Data Availability

Clinically interpreted variants and associated phenotypes from the GenROC study are available through DECIPHER (https://www.deciphergenomics.org/). Transcripts from interviews are not available due to the sensitivity of discussions and the risk to vulnerable children.
